# Lactate potentiates angiogenesis and neurogenesis in experimental intracerebral hemorrhage

**DOI:** 10.1038/s12276-018-0113-2

**Published:** 2018-07-06

**Authors:** Jing Zhou, Tao Liu, Hao Guo, Hanjin Cui, Pengfei Li, Dandan Feng, En Hu, Qing Huang, Ali Yang, Jun Zhou, Jiekun Luo, Tao Tang, Yang Wang

**Affiliations:** 1 Institute of Integrative Chinese Medicine, Xiangya Hospital, Central South University, 410008 Changsha, China; 2 Shanxi Province Hospital of Traditional Chinese Medicine, Shanxi Provincial Institute of Traditional Chinese Medicine, 030012 Taiyuan, China; 3 Department of Anesthesiology, Shanxi Provincial People’s Hospital, Affiliate of Shanxi Medical University, 030012 Taiyuan, China; 4 Department of Neurology, Xiangya Hospital, Central South University, 410008 Changsha, China; 5 Department of Neurology, Henan Province People’s Hospital, 450003 Zhengzhou, China; 6 Institute of Medical Sciences, Xiangya Hospital, Central South University, 410008 Changsha, China

**Keywords:** Regeneration and repair in the nervous system, Pathogenesis

## Abstract

Lactate accumulation has been observed in the brain with intracerebral hemorrhage (ICH). However, the outcome of lactate accumulation has not been well characterized. Here, we report that lactate accumulation contributes to angiogenesis and neurogenesis in ICH. In the first set of the experiment,  a rat model of ICH was induced by injecting collagenase into the brain. The effects of lactate accumulation on the neurological function, apoptosis, and numbers of newborn endothelial cells and neurons, as well as the proliferation-associated signaling pathway, were evaluated in the rat brain.  In the second set, exogenous l-lactate was infused into intact rat brains so that its effects could be further assessed. Following ICH, lactate accumulated around the hematoma; the numbers of PCNA^+^/vWF^+^ nuclei and PCNA^+^/DCX^+^ cells were significantly increased compared with the numbers in the Sham group. Moreover, ICH induced translocation of nuclear factor-kappa B (NF-κB) p65 into the nucleus, resulting in a notable upregulation of VEGF and bFGF mRNAs and proteins compared with the levels in the Sham controls. Administration of a lactate dehydrogenase inhibitor dramatically inhibited these effects, decreased the vascular density, and aggravated neurological severity scores and apoptosis after ICH. After exogenous l-lactate infusion, the numbers of PCNA^+^/vWF^+^ nuclei and PCNA^+^/DCX^+^ cells were strikingly increased compared with the numbers in the Sham controls. In addition, lactate facilitated NF-κB translocation to induce increased transcription of VEGF and bFGF. Co-infusion with an NF-κB inhibitor significantly inhibited these effects. These data suggest that lactate potentiates angiogenesis and neurogenesis by activating the NF-κB signaling pathway following ICH.

## Brain hemorrhage: Lactate aids in recovery

After a blood vessel bursts in the brain, lactate, a metabolic intermediary, accumulates around the bleeding site to increase new blood vessels and neurons. A team led by Tao Tang and Yang Wang at Central South University in Changsha, China, experimentally induced brain hemorrhage in rats and examined the effects of lactate in the brain. They showed that this metabolic signaling molecule protected against cell death and increased expression of growth factors involved in blood vessel formation and neural regeneration. Inhibiting lactate blocked these effects, while infusing exogenous lactate into the rat brains enhanced them. The findings suggest that lactate promotes angiogenesis and neurogenesis in experimental intracerebral hemorrhage.

## Introduction

Intracerebral hemorrhage (ICH) is one of the most devastating forms of stroke^1^. Only 12~39% of patients live independently after ICH, and the 30-day mortality of ICH is ~13.1–61%^2^. Metabolic changes in the brain are clearly a key element of ICH^3^. Previous studies have indicated that lactate, a metabolic intermediate, accumulates in the brain after ICH^4, 5^. Lactate was traditionally considered as an indicator of dysfunctional oxidative metabolism for neurons until Schurr et al. showed that lactate is able to support synaptic activity^6, 7^. Moreover, the astrocyte-neuron lactate shuttle hypothesis further verifies that lactate can be used by neurons^8^. In animal studies, the neuroprotective effect of lactate has been observed in a mouse model of cerebral ischemia^9^. In clinical studies, lactate administration improves the neurologic outcomes of traumatic brain injury patients^10^.

In addition to its neuroprotective effect, lactate has also been reported to be proangiogenic^11, 12^. Angiogenesis, an important process for the formation of new microvessels, is the essential endogenous mechanism of brain self-repair after ICH^13, 14^. Elevation of lactate in wounds enhances vascular endothelial growth factor (VEGF) synthesis^15^. Accumulated lactate in tumors, contributes greatly to the angiogenic phenotype through activation of nuclear factor-kappa B (NF-κB)^16^. Moreover, a recent study suggested that the implantation of a lactate-releasing biomimetic scaffold promoted vascularization and sustained neurogenesis in traumatic brain injury^17^. Lactate accumulation can be observed in the brain after ICH. However, the details of the impact of lactate following ICH have not been well characterized.

Angiogenesis and neurogenesis are vital brain repair processes after ICH, enhancing those processes may promote recovery^18^. VEGF, one of the most important proangiogenic growth factors, has been detected around the hematoma post-ICH^19^. Basic fibroblast growth factor (bFGF) activates endothelial cells (ECs) through fibroblast growth factor receptor-mediated signaling pathways^20^. Moreover, evidence suggests that VEGF and bFGF are important in neurogenesis and neuroprotection in the central nervous system^21, 22^. It has been demonstrated that NF-κB regulates the expression of numerous genes, including proliferation- associated VEGF and bFGF^23–25^. NF-κB is retained in the cytoplasm in an inactive state by binding to inhibitory IκB proteins. Once been activated, NF-κB translocates to the nucleus and activates its target genes^26^.

In the present study, we speculated that lactate could potentiate angiogenesis and neurogenesis in the rat brain following ICH by activating the NF-κB signaling pathway. These results may improve our understanding of events resulting from ICH and provide a novel therapeutic approach after ICH.

## Materials and methods

### Animals

Male Sprague-Dawley (SD) rats (220~250 g) were purchased from the Experimental Animal Center of Central South University (CSU). Rats were housed with access to food and water ad libitum under a 12-h light/dark cycle. The experiments were performed in compliance with the guidelines for the care and use of animals established by CSU and approved by the Institutional Animal Care and Use Committee of CSU (201403164).

### Establishment of ICH model

Rats were intraperitoneally anesthetized with 3% pentobarbital sodium (50 mg/kg). Rats were positioned in a stereotaxic frame (Stoelting Co., Chicago, IL, USA) and received an injection into the right globus pallidus (Collagenase, type VII, Sigma-Aldrich, USA, 0.5 U in 2.5 μL of 0.9% sterile saline). The coordinates of the injection were 1.4 mm posterior, 3.2 mm lateral to the bregma and 5.6 mm ventral to the cortical surface. The injection lasted for over 2 min, with the needle kept in position for an additional 10 min. In the Sham group, 2.5 μL of 0.9% sterile saline without collagenase was injected into the same site.

### Experimental design

There were two sets of experiments. In the first set, rats were subjected to collagenase-induced ICH. ICH rats were then randomly assigned to receive the lactate dehydrogenase (LDH) inhibitor oxamate (OXA, Sigma-Aldrich, artificial cerebrospinal fluid [aCSF], ALZET® Osmotic Pumps [0.5 µL/h], i.c.v.) at concentrations of 10, 25, or 50 mM. OXA was infused immediately after the collagenase injection. In the Sham group, rats only received 0.9% sterile saline and aCSF in the corresponding sites. Rats were killed on days 7 and 14 post-ICH. In the second set, intact rats received an infusion of sodium l-lactate (l-lactate, Sigma-Aldrich, 0.9% sterile saline, ALZET® Osmotic Pumps [0.5 µL/h]) into the right globus pallidus at concentrations of 5, 10, or 25 mM. Some rats in the l-lactate group received BAY11-7082 (BAY, Sigma-Aldrich, aCSF, 5 µL, i.c.v.) to inhibit NF-κB at concentrations of 25, 50, or 100 µM. BAY was injected immediately after the l-lactate-infusion with a cannula implantation system (RWD Life Science). BAY was injected once per day. In the Sham group, rats only received 0.9% sterile saline and aCSF in the corresponding sites. Rats were killed on days 2 and 7 after the l-lactate infusion.

### Behavioral test

The behavioral tests were evaluated by two investigators who were blinded to the experimental groups. The modified neurological severity score (mNSS) was evaluated. On days 1, 3, 7, and 14 after ICH, two observers scored the tests independently, and their scores were averaged.

### Sample preparation

Randomly chosen rats from each group were anesthetized with 3% pentobarbital sodium (50 mg/kg, i.p.). For morphological analysis, animals were transcardially perfused with 0.9% saline followed by ice-cold 4% paraformaldehyde. The removed brains were then postfixed in 4% paraformaldehyde for 4 h before dehydration and embedding with paraffin. For Western blot analysis, quantitative real-time polymerase chain reaction (qRT-PCR) and lactate concentration analyses, animals were perfused with only 0.9% saline, tissues adjacent to the hematoma were immediately stored in liquid nitrogen (to detect the expressions of nuclear/cytoplasmic NF-κB p65 in the Western blot analysis, fresh brain tissues were needed).

### Nissl’ staining

After deparaffinization, sections were washed in PBS and incubated in Nissl’ staining solution (Beyotime Biotechnology, China) for ~1 min. Sections were rinsed in PBS and dehydrated in gradient ethanol, cleared in xylene and covered with a coverslip. Quantitative survival neuron data were collected from independent fields of the perihematomal regions (400×).

### TUNEL staining

Apoptotic cells were detected in situ using the TUNEL kit (Roche, Germany). The TUNEL method was performed to visualize the 3ʹ-OH ends of DNA fragments in apoptotic cells. Samples were collected from the perihematomal regions (400×).

### Measurement of lactate concentration

The concentration of lactate in the perihematomal regions was determined using spectrophotometric and enzymatic methods. The tissues were homogenized in 0.9% sterile saline in an ice-bath (weight:volume = 1:9), the homogenate was centrifuged at 3000 rpm for 15 min at 4 °C. The lactate concentration was analyzed using a lactate assay kit (Nanjing Jiancheng Bioengineering Institute, China). Protein concentrations were measured using the bicinchoninic acid (BCA, Thermo Fisher) method. The lactate concentration is expressed as mmol/gprot.

### FITC–dextran labeling and microvessel density calculation

To assess the blood perfusion and vascular density of the microvascular system in the perihematomal regions of ICH, we injected FITC–dextran (molecular weight, 2 × 10^6^, Sigma-Aldrich, 50 mg/mL, 1 mL) into the tail vein for 1.5 min before rats were decapitated. The brains were rapidly removed and placed in 4% paraformaldehyde for 48 h at 4 °C. The brain sections (100-μm) were cut by using a vibratome and visualized with a laser confocal microscope (TCS SP8 X & MP, Leica). The percentage of the vascular area of the fluorescence signal composition of the entire vision field was measured (200×).

### Immunohistochemical analysis

Sections were immersed in 3% hydrogen peroxide for 15 min. Nonspecific antigen blocking was performed in 2% bovine serum albumin (BSA) for 1 h. Sections were then incubated overnight at 4 °C with mouse anti-VEGF (1:200, Abcam, UK), mouse anti-bFGF (1:200, Santa Cruz, USA), or rabbit anti-NF-κB p65 (1:400, Cell Signaling Technology, USA), then incubated with biotinylated anti-mouse IgG (1:800, Santa Cruz) or anti-rabbit IgG (1:800, Santa Cruz) for 1 h at 37 °C, and with the avidin-biotin-peroxidase complex (1:100, Vector Laboratories) for 1 h at 37 °C. Immunoreactivity was visualized with diaminobenzidine.

To detect proliferated cerebral microvascular ECs and neurons, we performed immunofluorescence double labeling. Sections were incubated in mouse anti-PCNA (1:1400, Cell Signaling Technology), with either rabbit anti-vWF (1:400, Dako, Denmark) or goat anti-DCX (1:100, Santa Cruz) for 1 h at 37 °C. To determine whether VEGF and bFGF were expressed in ECs or neurons, tissue sections were simultaneously incubated with mouse anti-VEGF (1:200, Abcam) or mouse anti-bFGF (1:100, Santa Cruz), and rabbit anti-vWF (1:400, Dako) or rabbit anti-NeuN (1:400, Cell Signaling Technology) for 1 h at 37 °C. The following secondary antibodies were then used: the Fluorescein 488-conjugated donkey anti-rabbit antibody (1:1000, Jackson Immunoresearch, USA), or Cy3-conjugated sheep anti-mouse antibody (1:1000, Jackson Immunoresearch). The visual data from the perihematomal regions were scanned using a laser confocal microscope.

### Western blot analysis

Tissues were homogenized in RIPA lysis buffer with a protease inhibitor. The homogenate was centrifuged at 12,000 rpm for 30 min at 4 °C. Protein concentrations were assayed with the BCA method. Proteins were segregated on SDS-PAGE gels and transferred onto PVDF membranes. The membranes were then blocked in 5% BSA for 2 h. The membranes were incubated with primary antibodies as follows: rabbit anti-IκBα (1:300, Santa Cruz), rabbit anti-p-IκBα (1:400, Millipore), mouse anti-VEGF (1:400, Abcam), mouse anti-bFGF (1:300, Santa Cruz), or mouse anti-β-actin (1:4000, Abcam) with gentle shaking at 4 °C overnight. Then, horseradish peroxidase-conjugated anti-mouse IgG (1:5000, Promega, USA) or anti-rabbit IgG (1:5000, Promega) secondary antibodies were incubated with the membranes for 2 h at room temperature. The immunopositive bands were visualized using an enhanced chemiluminescent substrate (Thermo Fisher) and Bio-Rad ChemiDoc XRS digital documentation system. The amount of protein expression is presented relative to the levels of β-actin. The nuclear/cytoplasmic proteins in fresh brain tissues were isolated using the Nuclear/Cytoplasmic Fractionation Kit (Beyotime Biotechnology). Nuclear/cytoplasmic proteins were used for NF-κB p65 detection; PVDF membranes were probed with primary rabbit anti-NF-κB p65 (1:1000, Cell Signaling Technology), mouse anti-β-actin (1:4000, Abcam) or rabbit anti-Histone H3 (1:2000, Cell Signaling Technology). The amount of cytoplasmic NF-κB p65 is presented relative to the levels of β-actin; nuclear NF-κB p65 is presented relative to the levels of Histone H3.

### qRT-PCR

Total RNA was obtained and purified using the E.Z.N.A. Total RNA Kit (Omega, USA); then, reverse transcription was performed with a reverse transcription assay kit following the manufacturer’s instructions (Applied Biosystems). Amplification was performed using SYBR Green All-in-oneTM qPCR Mix (GeneCopoeia) on a ViiA^TM^7 QRT-PCR system (Applied Biosystems). The following thermocycling protocol was used: 95 °C for 10 min, 40 cycles of 10 s at 95 °C, 50 s at 59 °C, and melting was done at 60 °C. The primers for VEGF, bFGF, and β-actin were designed with Premier 5.0 software for rats. The gene sequences of the primers are as follows: VEGF, 5′-TGGACCCTGGCTTTACTGCTG-3′ (forward) and 5′-GGCAATAGCTGC GCTGGTAGA-3′ (reverse); bFGF, 5′-GAACCGGTACCTGGCTATGA-3′ (forward) and 5′-CCGTTTTGGATCCGAGTTTA-3′ (reverse); β-actin, 5′-CATCCTGCGTCTG GACCTGG-3′ (forward) and 5′-TAATGTCACGCACGATTTCC-3′ (reverse). Melting curves of all of the samples were generated as controls for specificity. Expression data were normalized to the expression of β-actin with the 2^−ΔΔCt^ method.

### Statistical analysis

All data are expressed as the mean ± SD. Repeated-measures ANOVA (RM-ANOVA) was employed for behavioral tests. The remaining data were analyzed by using the Student *t*-test and one-way ANOVA. The criterion for statistical significance was *p* < 0.05. Statistical analyses were conducted using the SPSS 18.0 software package or Prism 5.0 (GraphPad).

## Results

### Lactate accumulated around the hematoma of ICH

**Fig. 1:**
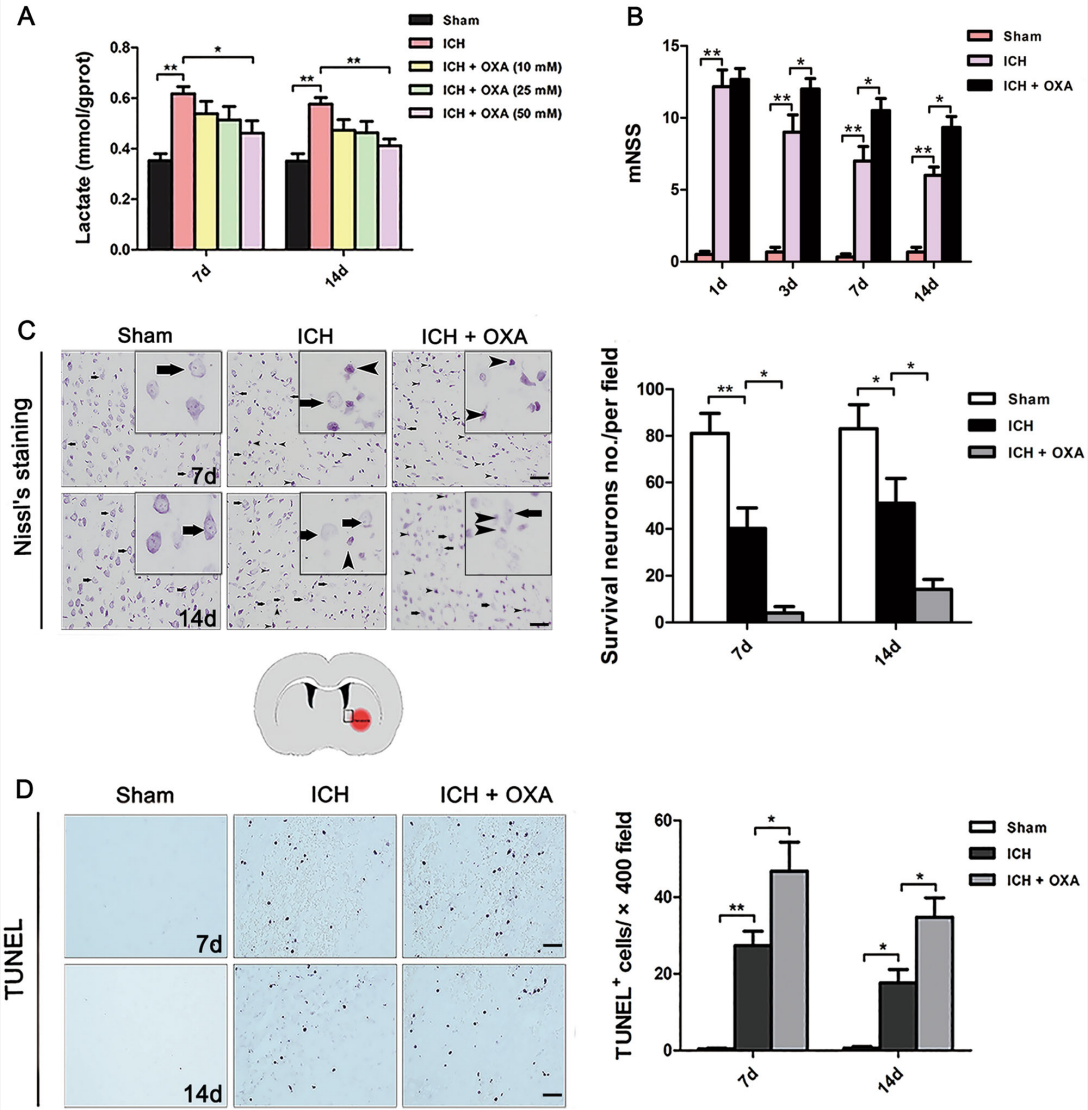
**a** Lactate production was significantly increased around the hematoma after ICH. OXA markedly lowered the lactate level (*n* = 5). **b** OXA-treated rats showed a significantly increased mNSS compared with that of the ICH group (*n* = 8). **c** Nissl staining showed that many neurons in the ICH group were damaged (arrows without tails); the number of surviving neurons (arrows with tails) was markedly reduced. In the OXA group, far fewer surviving cells were observed (*n* = 5). **d** TUNEL-positive cells markedly increased in abundance after ICH. In particular, the number of TUNEL-positive cells was significantly increased in the OXA-treated group after ICH (*n* = 5). ICH intracerebral hemorrhage, OXA oxamate. *p *<* 0.05 and **p *<* 0.01. Scale bar = 50 μm

The lactate content of perihematomal tissues was significantly increased at days 7 and 14 after ICH (Fig. 1a). We further found that the 50 mM OXA markedly suppressed the levels of lactate (Fig. 1a). Thus, in subsequent experiments, 50 mM OXA was used as the drug dose.

### Inhibition of endogenous lactate led to increased brain injury and severe neurological deficits after ICH

ICH rats exhibited neurological deficits on days 1–14 (Fig. 1b). On day 1, all rats subjected to ICH showed similar neurological results. Compared with the ICH group, OXA-treated rats exhibited more severe neurologic deficits on days 3, 7 and 14 after ICH, as demonstrated by an increase in mNSS (Fig. 1b).

Nissl’ staining was performed to evaluate neuron viability (Fig. 1c). The number of surviving neurons (Nissl bodies were stained evenly in the cytoplasm, with prominent nucleoli and loose chromatin; the cells were large with an abundant cytoplasm) was significantly reduced after ICH. After inhibition of endogenous lactate by OXA, far fewer surviving neurons were observed than in the ICH group (Fig. 1c). In TUNEL staining, few obvious apoptotic cells were detected in the brains of the Sham group on days 7 and 14 (Fig. 1d). Many TUNEL-positive cells appeared after ICH (Fig. 1d). OXA further aggravated apoptosis (Fig. 1d).

### Inhibition of endogenous lactate depressed angiogenesis, vascular density, and neurogenesis after ICH

**Fig. 2:**
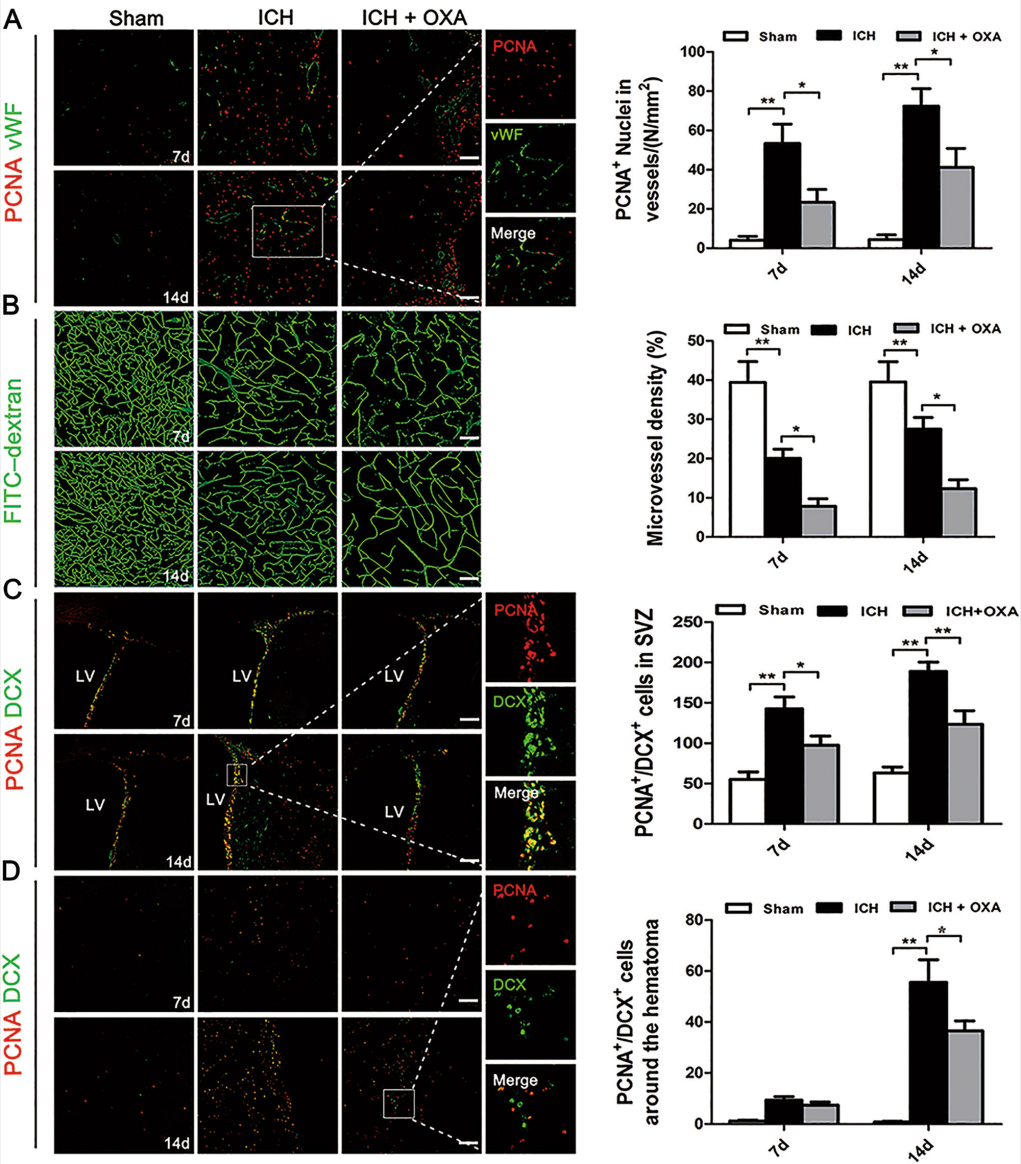
**a** Newborn nuclei in vWF^+^ vessels were present around the hematoma after ICH; OXA dramatically reduced PCNA^+^ nuclei in ECs. **b** In the ICH group, the brain vasculature was deformed and blood flow was obstructed. OXA further diminished the vascular density after ICH. **c**,**d** PCNA^+^/DCX^+^ cells were more prevalent after ICH than in the Sham group. OXA infusion notably decreased the numbers of PCNA^+^/DCX^+^ cells after ICH. ICH intracerebral hemorrhage; OXA oxamate. *p *<* 0.05 and **p *<* 0.01. *n* = 5. Scale bar = 100 μm

PCNA^+^ newborn nuclei were rarely observed in either hemisphere of the Sham group (Fig. 2a). The numbers of newborn nuclei in vWF^+^ dilated vessels appeared to increase on days 7 and 14 after ICH. OXA notably prevented the angiogenic effects (Fig. 2a). In the Sham group, brain microvessels were evenly distributed, with regular morphology, and the blood flow was unobstructed (Fig. 2b). ICH caused attenuated blood perfusion until day 14 (Fig. 2b). Vascular density was further diminished by OXA treatment (Fig. 2b).

Immunofluorescent double labeling of PCNA and DCX was applied to study the neurogenesis. Compared with the Sham group, PCNA^+^/DCX^+^ cells were more prevalent in the ipsilateral subventricular zone (SVZ) at days 7 and 14 (Fig. 2c), and were more prevalent in the perihematomal regions (Fig. 2d) at day 14 in ICH group. OXA significantly decreased the number of double-labeled cells (Fig. 2c, d).

### Lactate potentiated angiogenesis and neurogenesis

Given the evidence presented above, it was tempting to suppose that lactate accumulation potentiates angiogenesis and neurogenesis. To further verify our presumption, we directly infused l-lactate into intact rat brains. In the preliminary experiment, three dosages of l-lactate (5, 10, and 25 mM) were administered; rats were initially euthanized on the second day after injection. Immunofluorescence showed that lactate significantly increased the expression of PCNA^+^ nuclei in vWF^+^ dilated vessels around the l-lactate-affected region, the beneficial effects of 10 mM were more preeminent (Supplementary Figure S1A). Meanwhile, Western blot analysis of the VEGF and bFGF proteins showed similar results (Supplementary Figure S1B). Therefore, we infused l-lactate at a dose of 10 mM in the following experiments. On day 7, 10 mM l-lactate clearly promoted proliferation (Fig. 5d).

### Lactate promoted angiogenesis and neurogenesis by activating the NF-κB signaling pathway

**Fig. 3:**
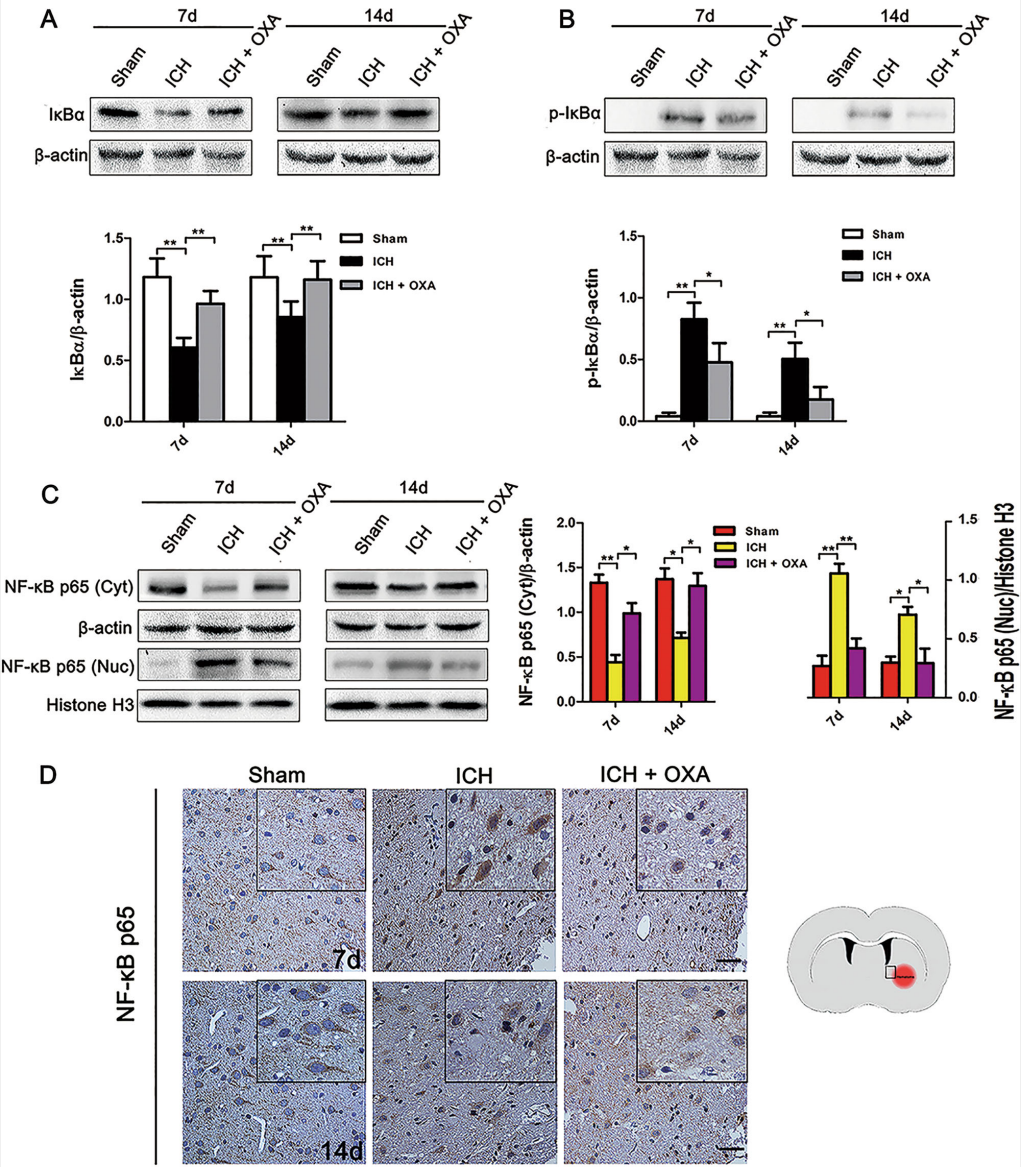
**a**,**b** Degradation and phosphorylation of IκBα occurred after ICH. OXA dramatically inhibited this effect. **c** Nuclear translocation of NF-κB p65 was significantly increased after ICH. This effect was significantly blocked by OXA. **d** Immunohistochemical analysis showed that NF-κB p65 was mainly retained in the cytoplasm in the Sham group, but some of the NF-κB p65 was transferred into the nucleus after ICH. ICH intracerebral hemorrhage, OXA oxamate. *p *<* 0.05 and **p *<* 0.01. *n* = 5. Scale bar = 50 μm

The protein expression of IκBα was significantly decreased with an increase in p-IκBα in the ICH group at days 7 and 14 compared with those of the Sham group (Fig. 3a, b). OXA caused marked dephosphorylation of IκBα (Fig. 3a, b). Moreover, translocation of NF-κB p65 into the nucleus occurred on days 7 and 14 after ICH (Fig. 3c). The translocation was significantly blocked by OXA (Fig. 3c). In the immunohistochemical analysis, NF-κB p65 was observed mainly in the cytoplasm in the Sham group (Fig. 3d), whereas NF-κB immunoreactivity translocated into the nucleus after ICH (Fig. 3d).

**Fig. 4:**
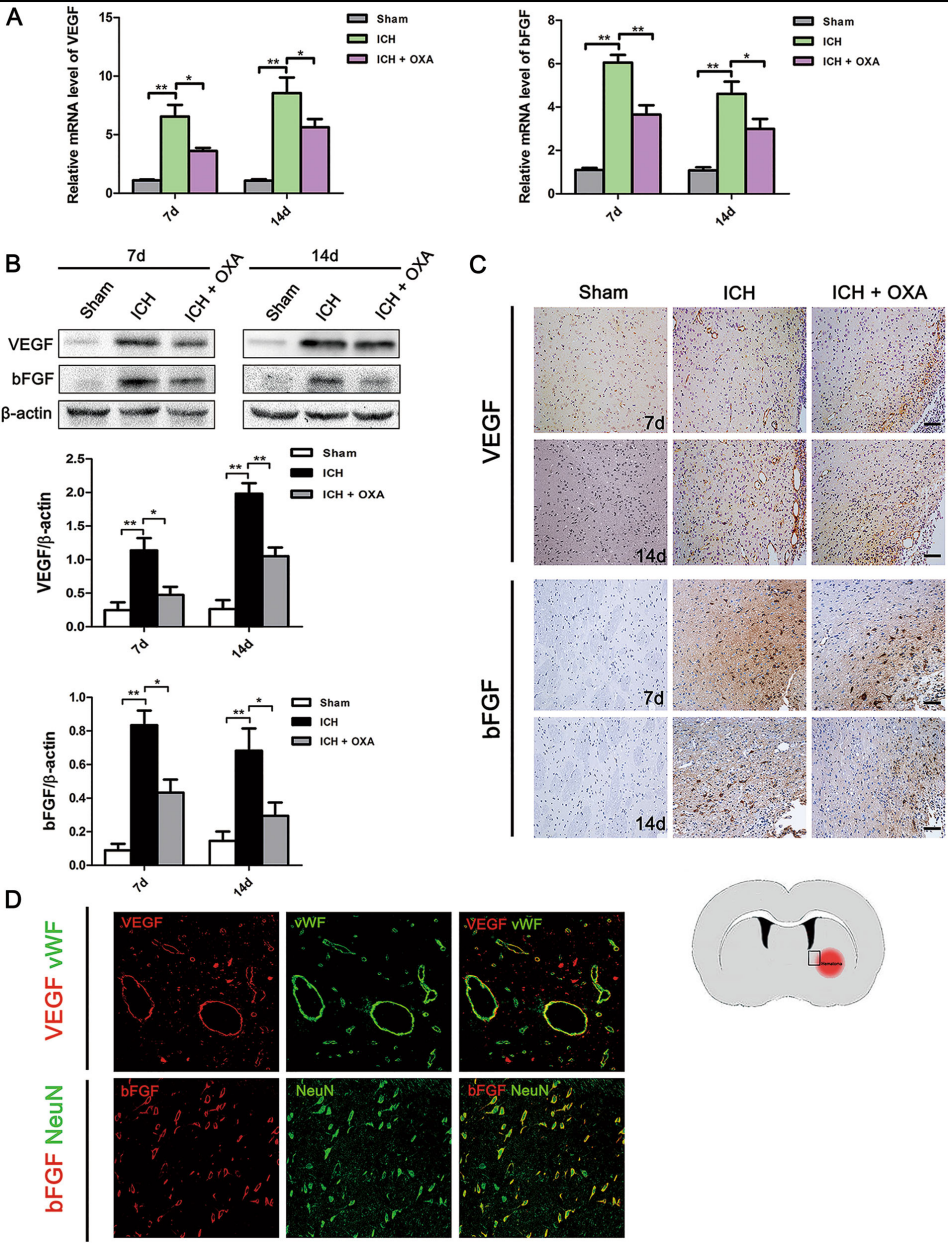
**a** Levels of VEGF and bFGF mRNAs were dramatically decreased in the OXA-treated group after ICH. **b** Western blot analysis showed that the expression levels of VEGF and bFGF were significantly upregulated after ICH. OXA suppressed this upregulation. **c** After ICH, many VEGF^+^ microvessels and bFGF^+^ cells were detected around the hematoma. **d** Immunofluorescent double labeling confirmed that VEGF was localized in vWF-positive vessels and that bFGF was localized to NeuN-positive cells. ICH intracerebral hemorrhage, OXA oxamate. *p < 0.05 and **p < 0.01. *n* = 5. Scale bar = 100 μm

Markedly increased VEGF and bFGF mRNA was detected at days 7 and 14 following ICH, OXA induced a significantly decrease in the expression of VEGF and bFGF mRNA (Fig. 4a). The Western blot analysis results demonstrated that the expression of the VEGF and bFGF proteins were significantly increased in the ICH group compared with those of the Sham group (Fig. 4b). OXA blocked these effects (Fig. 4b). Barely any VEGF or bFGF immunoreactivity was found in the Sham group (Fig. 4c). Many VEGF^+^ microvessels of the enlarged profile and bFGF^+^ cells were detected around the hematoma following ICH (Fig. 4c). Immunofluorescent double labeling confirmed that VEGF was localized to vWF-positive vessels and bFGF was localized to NeuN-positive cells (Fig. 4d).

**Fig. 5:**
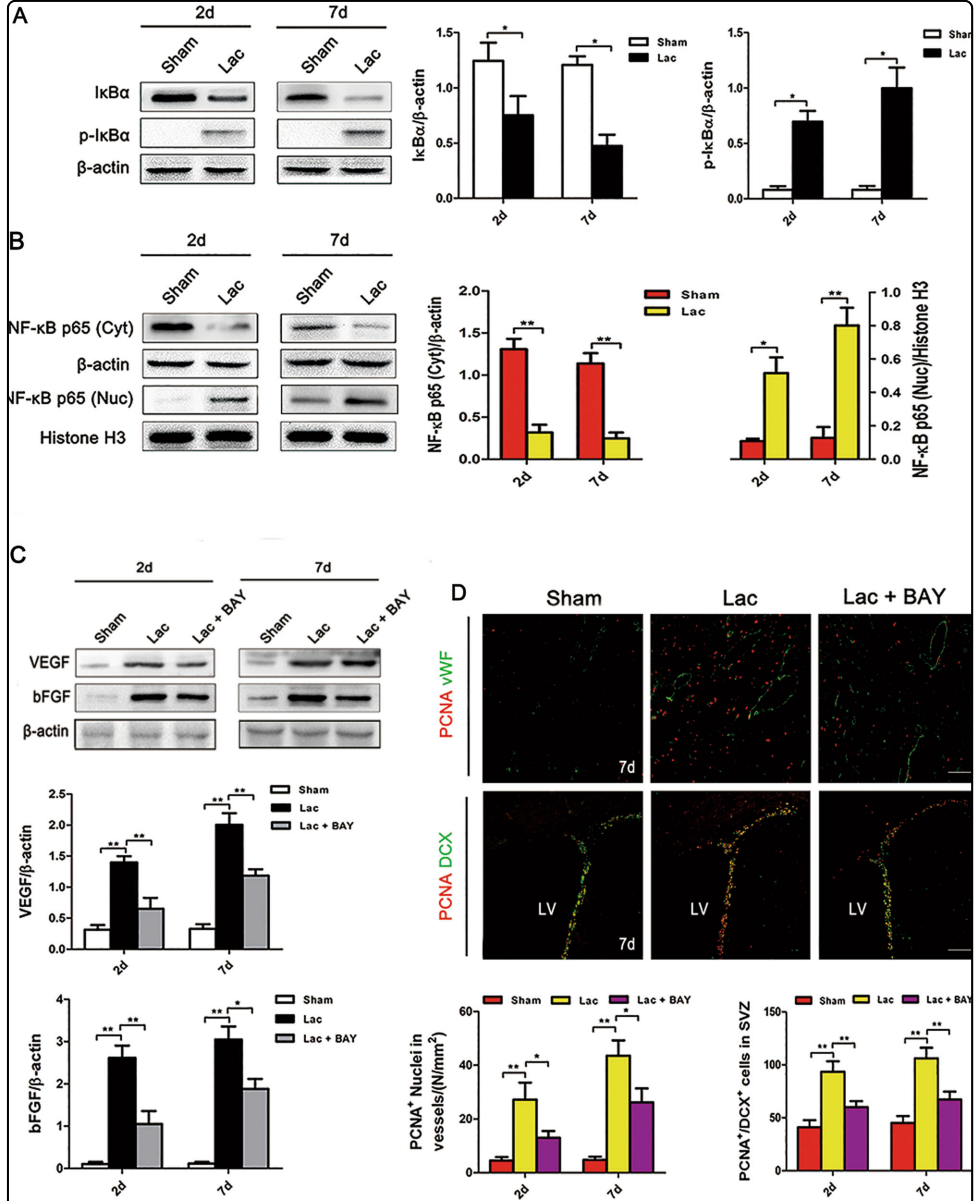
**a**,**b** IκBα was degradated and phosphorylated after l-lactate infusion, and NF-κB p65 protein translocated into the nucleus. **c** The expression levels of VEGF and bFGF were strikingly increased after l-lactate infusion. BAY blocked these effects. **d** l-lactate significantly increased the expression of PCNA^+^/vWF^+^ nuclei and PCNA^+^/DCX^+^ cells. BAY blocked these effects. Lac l-lactate, BAY BAY11-7082. *p *<* 0.05 and **p *<* 0.01. *n* = 5. Scale bar = 100 μm

As described above, OXA blocked activation of the NF-κB signaling pathway after ICH, indicating that the NF-κB signaling pathway played a vital role in lactate-mediated angiogenesis and neurogenesis. To test this hypothesis, we infused l-lactate into intact rat brains. Significant IκBα phosphorylation was observed on days 2 and 7 after l-lactate infusion (Fig. 5a). Moreover, nuclear NF-κB p65 protein was significantly increased after l-lactate infusion (Fig. 5b). The transcription levels of VEGF and bFGF were strikingly increased after lactate infusion (Fig. 5c). To further confirm our hypothesis, we injected BAY, an inhibitor of NF-κB, into some l-lactate-treated rats. In the preliminary experiment, 100 µM BAY exhibited the best inhibitory effect on day 2 after infusion (Supplementary Figure S2); thus, in the subsequent experiments, 100 µM BAY was applied. Western blot analysis showed that the expression levels of VEGF and bFGF were observably decreased after BAY intervention (Fig. 5c). Meanwhile, in the BAY-treated brains, lactate-induced expression of PCNA^+^ nuclei in vWF^+^ vessels and PCNA^+^/DCX^+^ cells was significantly decreased (Fig. 5d).

## Discussion

The major findings of the current study are as follows: (1) lactate accumulation was detected around the hematoma in ICH, (2) inhibiting endogenous lactate after ICH blocked ICH-induced angiogenesis and neurogenesis, (3) an exogenous lactate infusion promoted angiogenesis and neurogenesis, and (4) lactate-induced proliferation after ICH involved activation of the NF-κB signaling pathway. To the best of our knowledge, this is the first report on the protective and self-repair promoting effects of lactate in a rat model of ICH.

Following ICH, the resulting hematoma triggers a series of pathophysiological changes that lead to severe neurological impairments^27–29^. Here, OXA -treatment aggravated the neurologic deficits after ICH. LDH is an enzyme that catalyzes the conversion of pyruvate to lactate. OXA, as an inhibitor of LDH, abolishes the biological effects of endogenous lactate^30^. The results suggest that the accumulated lactate may have certain brain-protective effects in ICH. The subsequent Nissl staining and TUNEL assay results further supported this hypothesis.

### Lactate potentiates angiogenesis and neurogenesis in ICH

Angiogenesis involves the proliferation and migration of normally static ECs; it occurs at the highest levels from days 7 to 14 after ICH and reduces thereafter^31^. Angiogenesis provides nutritive blood flow for subsequent neurogenesis; the neurogenesis process also starts to increase at day 7 after ICH and is maintained at a high level until day 14^32^. Angiogenesis and neurogenesis are vigorous at day 7 and14 in the rat brain after ICH. Thus, days 7 and 14 were chosen to evaluate the pro-angiogenesis/pro-neurogenesis properties of lactate.

The pathological mechanism of ICH refers to inflammation, reactive oxygen species formation, and release of cytokines, proteases et al. Many factors can promote endogenous angiogenesis/neurogenesis after ICH. For example, thrombin, a serine protease that is released during hematoma formation after ICH, triggers angiogenesis/neurogenesis in rat brains that have undergone ICH^32, 33^. The establishment of new vessels and the potential for neural regeneration hold promise for vascular perfusion, energy supply and brain self-repair. In the present study, we detected numerous newborn microvessels around the hematoma. Moreover, neurogenesis was also observed. All of these results are consisted with previous studies^32, 33^. We showed that OXA markedly inhibited lactate levels and simultaneously attenuated ICH-induced vascular and neuronal proliferation. These results indicate that lactate is a direct endogenous angiogenic and neurogenic mediator in the brain following ICH. The results of the intracerebral infusion of l-lactate further supported our presumption.

### Lactate promotes angiogenesis and neurogenesis by activating the NF-κB signaling pathway

Inhibition of endogenous lactate accumulation blocked the activation of NF-κB p65 after ICH. In addition, our data showed that an exogenous l-lactate infusion activated NF-κB p65. BAY is an NF-κB inhibitor^34, 35^, after co-infusion of lactate and BAY we observed that it inhibited the NF-κB-dependent VEGF and bFGF expression levels and depressed newborn ECs and neurons. All these results indicate that lactate triggers angiogenesis and neurogenesis via the NF-κB signaling pathway after ICH. In response to lactate stimulation, the NF-κB repressor IκB is phosphorylated and degraded, and the dissociative NF-κB subsequently translocates into the nucleus and exposes the nuclear localization signals on the p50/p65 heterodimer, which leads to VEGF and bFGF transcription. Clarification of the mechanism by which lactate promotes angiogenesis and neurogenesis may provide new therapeutic opportunities for ICH. Despite having shown the protective effects of lactate, the present study did not determine the detailed mechanisms of lactate accumulation and transport after ICH. A previous study has demonstrated that lactate can induce a phenotype shift in macrophages, as well as expression of VEGF, which promotes proliferation^36^. Following ICH, there are many microglia/macrophage cells around the hematoma region, and further research is needed to explore whether immune cells or other mechanisms play related roles in the lactate-induced proliferative effect following ICH. Additionally, monocarboxylate transporters (MCTs) are essential for the usage of lactate^37^ the possible role of MCTs in lactate transportation and angiogenesis/neurogenesis after ICH also need to be explored.

**Fig. 6:**
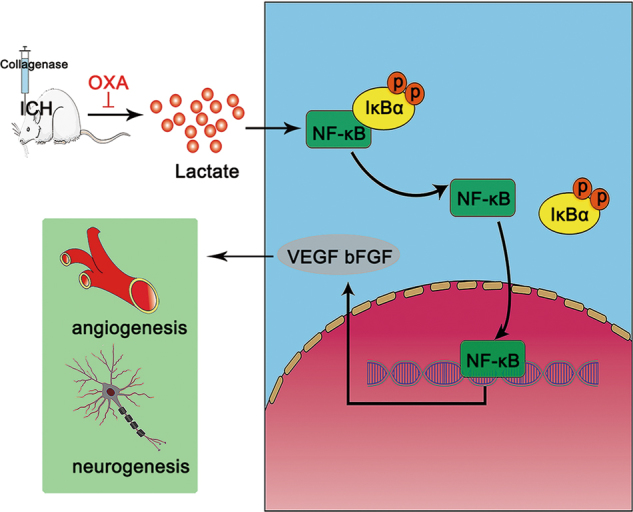
Lactate potentiates angiogenesis and neurogenesis by activating the NF-κB signaling pathway following ICH

In conclusion, we demonstrate that ICH-induced lactate accumulation can effectively protect against cell apoptosis and simultaneously promote angiogenesis and neurogenesis. Moreover, the mechanism whereby lactate promotes angiogenesis and neurogenesis may rely on activation of the NF-κB signaling pathway (Fig. 6). In order for the research results to be more clinically relevant or translational in the near future, we will try to determine whether there is any correlation between the serum and brain lactate levels in ICH rats; we will also address the possibility of translating our findings to ICH patients.

## Electronic supplementary material

Supplementary materials
