# Supplementary material for: Lactate potentiates angiogenesis and neurogenesis in experimental intracerebral hemorrhage
Source: Exp Mol Med. 2018 Jul 6;50(7):1–12. doi: 10.1038/s12276-018-0113-2 (PMC6035243; doi:10.1038/s12276-018-0113-2)
Supplement: Supplementary file 1 — Supplementary materials [file 12276_2018_113_MOESM1_ESM.pdf]

Supplementary materials

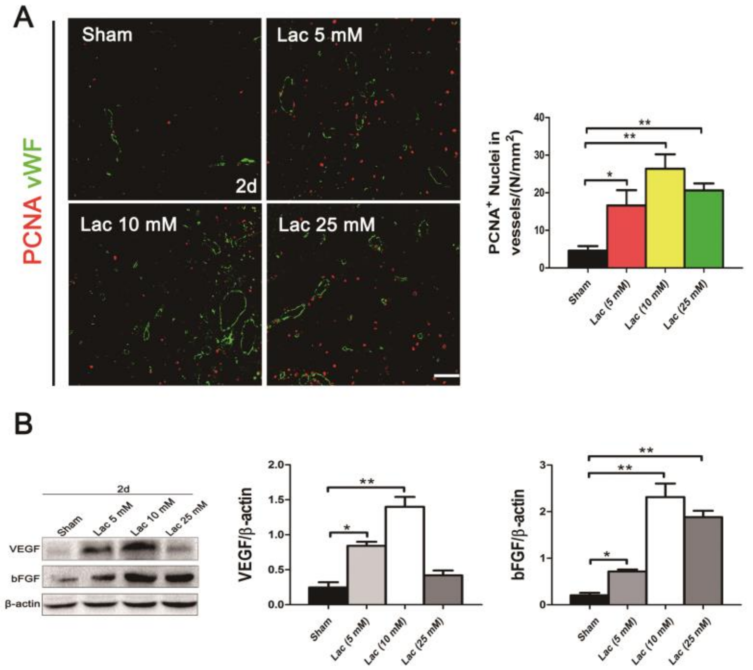

Supplementary Figure S1. (A) The numbers of PCNA$^+$ nuclei in vWF$^+$ dilated vessels were rarely observed in either hemisphere of the Sham controls on the day 2. Notably, PCNA$^+$ nuclei in vWF$^+$ dilated vessels appeared to be present around the L-lactate (5, 10 and 25 mM) - affected regions. **(B)** The expressions of VEGF were strikingly increased after 5 and 10 mM L-lactate infusion. The expressions of bFGF were strikingly increased after L-lactate infusion (5, 10 and 25 mM). The beneficial effects of 10 mM were more preeminent. Lac: L-lactate. (n=5, **p* <0.05 and ***p* <0.01).

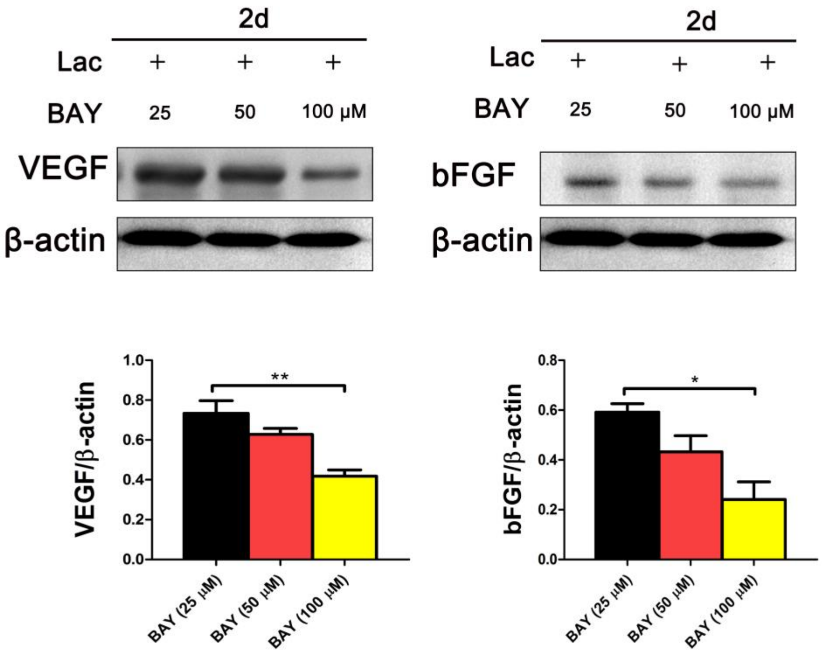

Supplementary Figure S2. Western blot showed that BAY inhibited the expressions of VEGF and bFGF in L-lactate group. The effects of 100 μM BAY were most obvious. Lac: L-lactate; BAY: BAY11-7082. (n=5, **p* <0.05 and ***p* <0.01).

.
